# Histological comparison between preoperative and surgical specimens of non-small cell lung cancer for distinguishing between "squamous" and "non-squamous" cell carcinoma

**DOI:** 10.1186/1746-1596-9-103

**Published:** 2014-05-29

**Authors:** Tomoko Yamagishi, Katsuhiko Shimizu, Nobuaki Ochi, Hiromichi Yamane, Isao Irei, Yoshito Sadahira, Nagio Takigawa, Mikio Oka, Masao Nakata

**Affiliations:** 1 Department of General Internal Medicine 4, Kawasaki Medical School, 2-1-80, Nakasange, Kita-ku, Okayama 700-8505, Japan; 2 Department of General Thoracic Surgery, Kawasaki Medical School, 577 Matsushima, Kurashiki, Okayama 701-0192, Japan; 3 Department of Pathology, Kawasaki Medical School, 577 Matsushima, Kurashiki, Okayama 701-0192, Japan; 4 Department of Respiratory Medicine, Kawasaki Medical School, 577 Matsushima, Kurashiki, Okayama 701-0192, Japan

## Abstract

**Background:**

Non-small cell lung cancers (NSCLCs) are frequently heterogeneous and in approximately 70% of cases, NSCLCs are diagnosed and staged by small biopsies or cytology rather than by examination of surgically resected specimens. Thus, in most patients, the diagnosis is established based on examination of preoperative specimens alone. Recently, classification of NSCLC into pathologic subtypes has been shown to be important for selecting the appropriate systemic therapy, from both the point of view of treatment efficacy and prevention of toxicity.

**Methods:**

We retrospectively reviewed the data of 225 patients to compare the preoperative classification of the NSCLC subtype on biopsy specimens with the postoperative classification based on examination of the resected specimens, in order to compare the accuracy of the two for the diagnosis of various histological subtypes of NSCLC.

**Results:**

In 169 of the 225 (75.1%) patients, the preoperative diagnosis was definite malignancy. Histologically, the final pathologic diagnosis made from the surgical specimens was adenocarcinoma (ADC) in 169 patients, and in 75.5% of these cases, the diagnosis was concordant with the preoperative diagnosis. Among the patients who had squamous cell carcinoma (SQC) in the preoperative specimens, the diagnosis was concordant with the preoperative diagnosis in 65.7% of cases. Misclassified preoperative biopsies included an even number of SQCs and ADCs, with all the misclassified biopsies being ADCs morphologically mimicking SQC due to solid growth. Significantly higher specificity, negative predictive value and accuracy were observed for the diagnosis of SQC.

**Conclusions:**

Our study suggested that the concordance rates for diagnosis of the NSCLC subtypes, especially the "squamous" or "non-squamous" histologies, between preoperative and surgical specimens were satisfactory, as compared with previous reports. Therefore, pretreatment diagnosis of lung cancer using small samples is reasonable for selecting the optimal treatment. However, in order not to lose the opportunity for selecting an effective treatment, we should be aware that the diagnosis in preoperative small samples might be different from that based on examination of the surgical specimens.

**Virtual Slides:**

The virtual slide(s) for this article can be found here: http://www.diagnosticpathology.diagnomx.eu/vs/2032698427120488

## Background

Lung cancer is the leading cause of cancer and mortality from cancer worldwide [1], with 5-year survival rates of <15% across all stages of the disease [2]. Historically, lung cancer is divided into two morphologic types: small cell lung cancer and non-small-cell lung cancer (NSCLC), with NSCLC accounting for approximately 80% to 85% of all histological types of NSCLC [3].

The International Association for the Study of Lung Cancer, the American Thoracic Society, and the European Respiratory Society recently proposed the classification of NSCLC based on examination of small biopsy and/or cytology specimens into six main subtypes: adenocarcinoma (ADC), squamous cell carcinoma (SQC), NSCLC not otherwise specified (NSCLC-NOS), NSCLC with neuroendocrine morphology, NSCLC with squamous and adenocarcinoma patterns, and poorly differentiated NSCLC with spindle and/or giant cell carcinoma [4]. NSCLCs are frequently heterogeneous and approximately 70% of NSCLCs are diagnosed and staged by examination of small biopsy or cytology specimens rather than by examination of surgically resected specimens [5]; thus, in most patients, the diagnosis is based on examination of preoperative specimens alone. Until now, histological subtyping of NSCLC was not considered to be clinically or therapeutically important, because of the lack of existence of differential treatment options for the various subtypes of NSCLC. However, there is now clear evidence to suggest that the classification of NSCLC into pathologic subtypes is important for the selection of appropriate systemic therapy, from both the point of view of treatment efficacy and prevention of toxicity [6,7]. For example, large phase III clinical trials have demonstrated that ADC is a strong predictive factor for improved outcome following treatment with the combination of pemetrexed with cisplatin compared with SQC [8]. In addition, the presence of common activating mutations in the tyrosine kinase domain of the epidermal growth factor receptor (*EGFR*) gene (exon 19 deletion and exon 21 L858R mutation) confers strong sensitivity to gefitinib and erlotinib, which are selective tyrosine kinase inhibitors of *EGFR*[9,10]. These *EGFR* mutations are more commonly encountered in ADCs [11]. Furthermore, the use of vascular endothelial growth factor (VEGF) inhibitors (eg, bevacizumab) has been demonstrated to be associated with an increased risk of fatal pulmonary hemorrhage in patients with SQC [12]. The *ALK* tyrosine inhibitor crizotinib was demonstrated to show marked antitumor activity in NSCLC patients with *EML4-ALK* translocation, observed in approximately 3 to 4% of patients with ADC [13]. Thus, a molecular testing guideline has been proposed [14].

If pathological findings are missed, especially when differentiating between "squamous" and "non-squamous" histology, determination of the appropriate treatment is difficult. The aim of this study was to compare the preoperative classification of NSCLC based on biopsy specimens with the postoperative classification made on the basis of examination of resected specimens, and to examine how often accuracy about each histological subtypes of NSCLC.

## Methods

### Patient population

Between April 2004 and June 2011, 442 patients underwent surgical resection for lung cancer at Kawasaki Medical School Hospital, Kurashiki, Japan. We retrospectively reviewed the data of a consecutive series of 225 (50.9%) patients in whom preoperative diagnosis was made by transbronchial biopsy (TBB) or computed tomography-guided fine needle biopsy (CTNB). For each patient, the clinical information at diagnosis was collected from the medical records. This study was conducted with the approval of the institutional Ethics Committee of Kawasaki Medical School (No.887).

### Transbronchial biopsy and computed tomography-guided fine needle biopsy procedures

While TBB is commonly performed in the diagnostic workup of pulmonary peripheral nodules, CTNB is usually performed either when the tumor is not detected bronchoscopically or when it is thought unlikely to be accessible by bronchoscopy. Flexible bronchoscopy is conducted by experienced bronchoscopists, while CTNB is performed by interventional radiologists, with standard techniques for both. All aspirated materials and biopsy specimens were fixed in formalin and embedded in paraffin, and sections are routinely stained with hematoxylin and eosin (H&E). Cytological examinations of sputum, aspirated material and bronchial brushings, and washing were not included in the diagnostic workup in this study, because the aim of the study was histological comparison between preoperative biopsy specimens and surgically resected specimens.

The results of the examination of the small diagnostic specimens were classified as no malignancy, suspected malignancy or definite malignancy. Furthermore, definite malignancy of NSCLC was classified into ADC, SQC, NSCLC or other histological subtypes (others). If there is no clear ADC or SQC morphology, the tumor can be further classified based on immunohistochemocal stains (IHC) and mucin (periodic acid Schiff) stains. If the stains all favor ADC: positive ADC markers (i.e., TTF-1 and/or mucin positive) with negative SQC markers, then the tumor is classified as ADC. If SQC markers (i.e., p63) are positive with negative ADC markers, the tumor is classified as SQC. If the ADC and SQC markers are both strongly positive and negative, the tumor is classified as NSCLC-NOS and others.

### Statistical analysis

Sensitivity, specificity, positive predictive value (PPV), negative predictive value (NPV), and accuracy were calculated according to the standard definitions for the diagnosis of lung cancer and malignancy. Continuous variables were analyzed by the Student's *t-*test, and the results were expressed as mean ± standard deviation (SD). Dichotomous variables were analyzed by the Fisher's exact test or the χ^2^ test, as appropriate. Discontinuous variables were coded as dummy variables. Two-sided p-values of less than 0.05 were considered to be statistically significant. All analyses were performed using the SPSS software (Version 17.0; SPSS Incorporation, Chicago, IL).

## Results

### Case report

**Figure 1:**
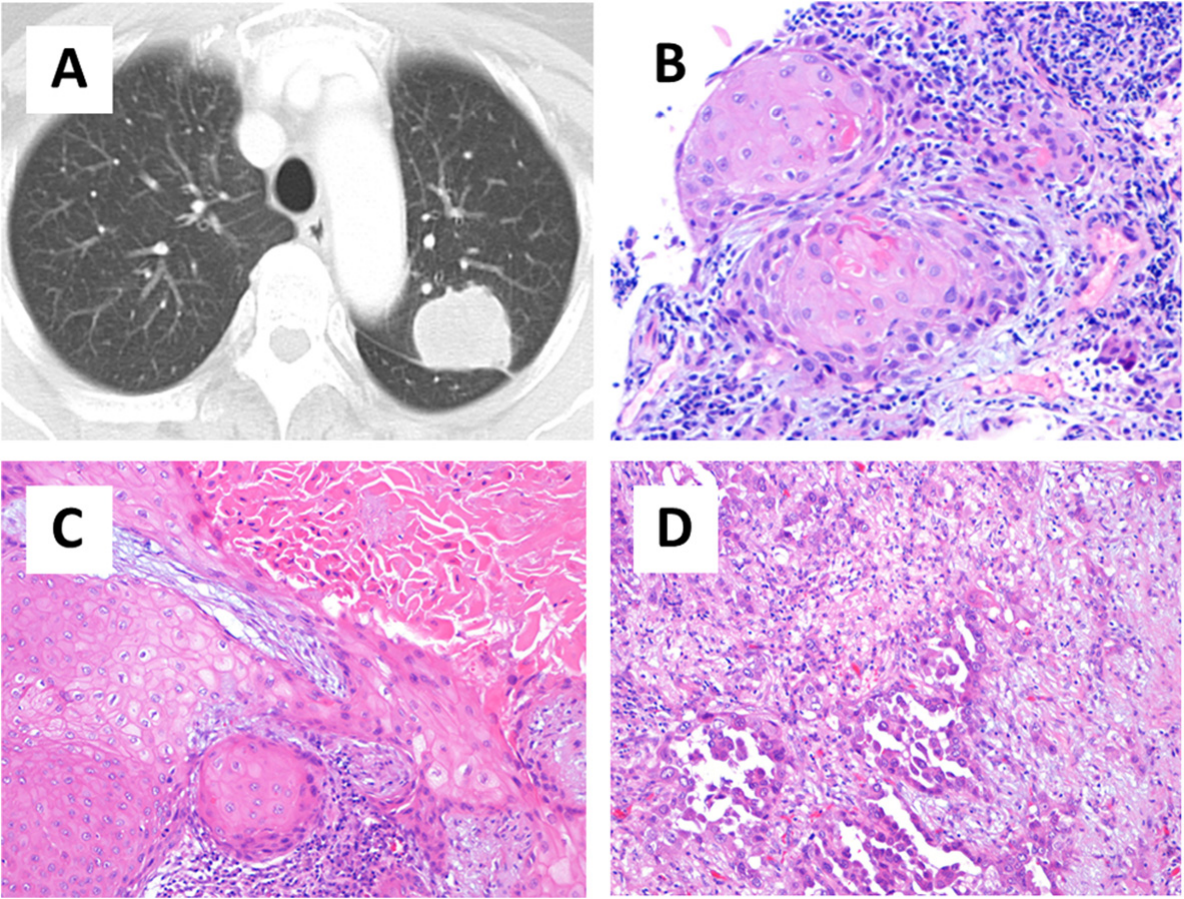
**A case with discordant diagnosis of the histological subtype between preoperative and surgical specimens. (A)** Computed tomographic image showing a mass lesion measuring 4 cm in diameter in the left upper lobe. **(B)** Computed tomography-guided fine needle biopsy was performed. Pathological findings of the biopsy specimen showed keratinized cells, suggestive of squamous cell carcinoma (H.E. stain, ×100). **(C-D)** Surgically resected specimen showing both a squamous and glandular component, suggesting the postoperative diagnosis of adenosquamous carcinoma (H.E. stain, ×100).

A 74-year-old woman who was a never-smoker was referred to our hospital because of hoarseness. Computed tomography showed a solid mass approximately 4 cm in diameter in the left upper lobe associated with mediastinal lymphadenopathy (Figure 1A). CTNB was carried out, and biopsy examination confirmed a well-keratinized tumor without obvious glandular features or cytoplasmic mucin (Figure 1B). The patient was diagnosed as having SQC of the lung, stage IIIA (T2aN2M0). Left upper lobectomy with mediastinal lymph node dissection was performed. Both ADC and SQC components were observed in the pathological specimen, suggesting the postoperative diagnosis of adenosquamous carcinoma (Figure 1C&D). One year later, recurrence was found in the form of mediastinal lymphadenopathy. We performed a molecular analysis of the surgical specimens, which revealed an *EGFR* gene mutation, and treatment with oral gefitinib was initiated. The patient would have lost the opportunity to receive gefitinib treatment if had just been diagnosed as having inoperable advanced NSCLC, because the molecular testing guideline does not recommend *EGFR* mutation analysis in patients with SQC [14].

### Patient characteristics

A total of 225 patients (159 men and 66 female; mean age, 69.0 ± 10.2 years) were enrolled in this study. The pathological stage and histological type on the final pathological examination are shown in Table 1. The most frequent histological type was ADC; 139 (61.5%) had ADC, 67 (30.1%) had SQC, 10 (4.4%) had large cell carcinoma, and 9 (4.0%) had others (other subtypes). The patients were classified according to the histopathological stage as follows: 131 patients had stage I, 47 had stage II, and 47 had stage III or IV disease. Most stage III and IV patients were recognized during and after surgery due to mediastinal lymph node metastasis (cN0-pN2) and pleural dissemination (cM0-pM1a). A few stage III patients were performed resection due to single mediastinal lymph node metastasis.

**Table 1:** Patient characteristics (n = 225)

**Variable**	**Number**	**%**
Age, mean ± SD	69.0 ± 10.2	
Sex		
Male	159	70.7
Female	66	29.3
Histology		
Adenocarcinoma	139	61.5
Squamous cell	67	30.1
NSCLC-NOS	10	4.4
Pleomorphic	5	2.2
Adenosquamous	4	1.8
Pathological stage		
IA	78	34.5
IB	53	23.9
IIA	23	10.2
IIB	24	10.6
IIIA	36	15.9
IIIB/IV	11	4.9

*Abbreviations*: *NSCLC-NOS* Non–small-cell lung carcinoma not otherwise specified.

### Sensitivity of preoperative diagnosis for the detection of NSCLC

The preoperative diagnosis was compared with the final pathological findings (Table 2). In 169 of the 225 (75.1%) patients, the preoperative diagnoses were definite malignancy (115 ADC, 45 SQC, 2 large cell carcinoma, 2 pleomorphic carcinoma, and 5 NSCLC). In 56 of the 225 (24.9%) patients, the preoperative specimens showed suspected or no malignancy: while NSCLC was suspected preoperatively in 13 of these patients, no malignancy was suspected in 43 patients. The overall sensitivity for the detection of lung cancer was 75.1%.

**Table 2:** Comparison between preoperative specimens and surgical diagnosis

**Preoperative diagnosis**	**Postoperative diagnosis**					
	**ADC**	**SQC**	**NSCLC-NOS**	**ASC**	**PLE**	**Total**
Malignant						
ADC	105	3	2	3	2	115
SQC		44		1		45
NSCLC-NOS	2	1	4			7
PLE					2	2
Suspicious of NSCLC	5	7	1			13
Not malignant	27	12	3		1	43
Total	139	67	10	4	5	225

*Abbreviations*: *ADC* adenocarcinoma, *SQC* squamous cell carcinoma *NSCLC-NOS* non-small-cell lung cancer not otherwise specified, *ASC* adenosquamous carcinoma, *PLE* pleomorphic carcinoma.

Histologically, the final pathological examination of the surgical specimens revealed ADC in 139 patients, of whom in 105 (75.5%) patients, the diagnosis was concordant with the preoperative diagnosis. Of the remaining 34 patients, 2 patients had NSCLC, 5 patients had suspected NSCLC, and 27 patients had no malignancy. In all, 67 patients had SQC of the lung on the preoperative specimens, and in 44 (65.7%) of these patients, the diagnosis was concordant with the preoperative diagnosis. Among the 23 of the remaining 67 (34.3%) with a discordant diagnosis, 3 patients had ADC, 1 patient had NSCLC, 7 patients had suspected NSCLC and 12 patients had no evidence of malignancy. Misclassified preoperative biopsies included an even number of SQCs and ADCs (n = 3), whereas all of the misclassified biopsies were ADC, morphologically mimicking SQC due to the solid growth.

We compared the sensitivity, specificity, predictive values and accuracy for the diagnosis of ADC and SQC (Table 3). Significantly higher specificity, NPV and accuracy were observed for SQC (p < 0.05).

**Table 3:** Diagnostic value of preoperative diagnosis between adenocarcinoma and squamous cell carcinoma

	**Sensitivity**	**Specificity**	**PPV**	**NPV**	**Accuracy**
	**(%)**	**(%)**	**(%)**	**(%)**	**(%)**
**Lung cancer**	75.1	-	100	100	75.1
	(169/225)		(169/169)	(56/56)	(169/225)
ADC	75.5	88.3	91.0	69.0	80.4
	(105/139)	(76/86)	(105/115)	(76/110)	(181/225)
SQC	65.6	99.3	97.7	87.2	89.3
	(44/67)	(157/158)	(44/45)	(157/180)	(201/225)
p-value (ADC vs. SQC)	0.138	<0.001	0.184	<0.001	0.012

*Abbreviations*: *PPV* Positive predictive value, *NPV* Negative predictive value, *ADC* adenocarcinoma, *SQC* squamous cell carcinoma.

### Comparison of H&E stain vs. IHC for subtyping NSCLC

**Figure 2:**
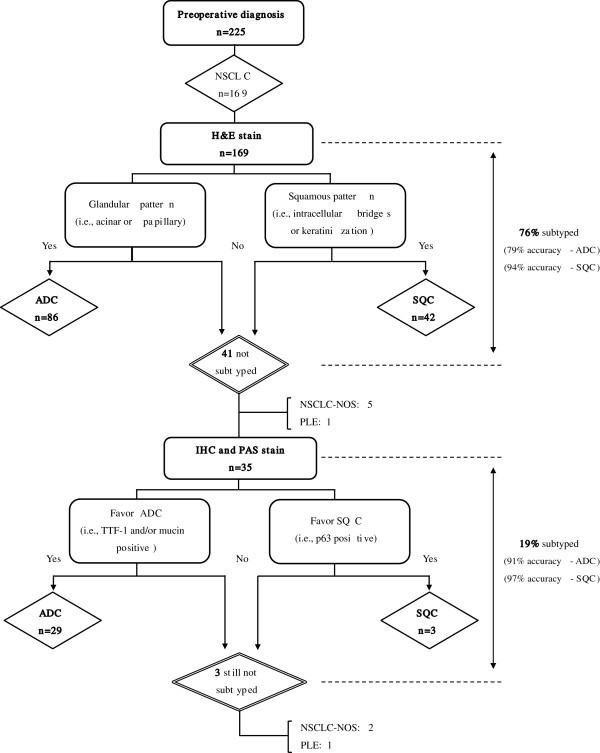
Diagnostic algorism for classification of NSCLC in preoperative specimens.

For the comparison of H&E stain vs IHC, 169 patients were included, all of whom underwent H&E stain. Among them, IHC was performed on 35 (21%) patients (Table 4). As summarized in Figure 2, 128 of 169 patients (76%) were specifically subtyped on the H&E stain as either ADC or SQC. 41 (24%) patients were not subtyped on the H&E stain sections. IHC could successfully subtype NSCLC in 32 of 35 cases (29 ADC, 3 SQC). The remaining three cases were not still subtyped (2 NSCLC-NOS, 1 PLE). However, by combining the results of the two methods, the number of patients with not subtyped diagnoses was reduced to 9 (5%) patients. The accuracy of both morphologic differentiation and IHC based predictive subtype is <100% (Figure 2).

**Table 4:** Comparison of H&E stain vs IHC for subtyping NSCLC

	**Preoperative diagnosis**							
**Postoperative diagnosis**	**H&E diagnosis**				**IHC predicted phenotype**			
	**ADC**	**SQC**	**Unclassified**	**Total**	**ADC**	**SQC**	**Unclassified**	**Total**
ADC	79		28	107	26			26
SQC	2	40	6	48	1	3		4
Unclassified	5	2	7	14	2		3	5
Total	86	42	41	169	29	3	3	35

*Abbreviations*: *ADC* adenocarcinoma, *SQC* squamous cell carcinoma, *NSCLC-NOS* non-small-cell lung cancer not otherwise specified, *PLE* pleomorphic carcinoma, *H&E* hematoxylin and eosin, *IHC* immunohistochemistry.

### Comparison of TBB vs. CTNB for the sensitivity or concordance of lung cancer diagnosis

Among the 225 patient studied, TBB was performed in 190 patients. Of these, 140 (73.7%) were diagnosed as having definite lung cancer, 13 (6.8%) were suspected as having malignancy, and 37 (19.5%) were diagnosed as having no malignancy. CTNB was performed in 35 patients. Of these, 29 (82.9%) and 6 (17.1%) cases were diagnosed as having lung cancer and no malignancy, respectively. Thus, there was no significant difference in the level of diagnostic certainty between TBB and CTNB (Table 5).

**Table 5:** Comparison of TBB vs. CTNB for sensitivity/concordance rate

	**Histological diagnosis**						
	**Lung cancer**	**Suspicious of malignancy**	**No malignancy**	**Sensitivity**	**p-value**	**Concordance**	**p-value**
TBB	140	13	37	73.2 (140/190)	0.249	66.8 (127/190)	0.594
CTNB	29	0	6	82.9 (29/35)		71.4 (25/35)	
Total	169	13	43	75.0 (169/225)		67.6 (152/225)	

*Abbreviations*: *TBB* Transbronchial biopsy, *CTNB* Computed tomography-guided fine needle biopsy.

### Comparison of the sensitivity or concordance rate of the histological type between preoperative and surgical specimens in relation to the tumor size

The results of detection of lung cancer were correlated with the tumor size. The overall sensitivity and concordance rate of histological type between preoperative biopsies and surgical specimens were 75.1% (169/225) and 67.6% (152/225). The sensitivity rate was correlated with the tumor size. The diagnostic sensitivity of lesions larger than 2.1 cm in diameter was significantly better than that of lesions less than 2 cm in diameter (p = 0.001, Table 6). However, concordance rate was not correlated with the tumor sizes.

**Table 6:** Comparison between tumor size and sensitivity/concordance rate

	**Histological diagnosis**						
	**Lung cancer**	**Suspicious of malignancy**	**No malignancy**	**Sensitivity**	**p-value**	**Concordance**	**p-value**
<=2 cm	30	0	19	61.2 (30/49)	0.011	57.1 (28/49)	0.078
>2 cm	139	13	24	78.9 (139/176)		70.4 (124/176)	
Total	169	13	43	75.0 (169/225)		67.6 (152/225)	

## Discussion

We assessed the accuracy of diagnosis of the histological type of lung cancer between preoperative and surgical specimens. The reported accuracy for bronchoscopic biopsy as compared to histopathology of resected or autopsy specimens for differentiating among histological subtypes of lung cancer is in the range of 62% to 97.5% [15-19]. In our study, the concordance rates for diagnosis of the NSCLC subtypes between preoperative biopsies and histopathology of surgical specimens was 66.8% (127/190), which seemed to be slightly low as compared to previous reports; however, many studies excluded tumors that were confirmed as not being malignant: in our study, 50 (26.3%) of 190 patients were diagnosed as not having malignant lesions before excision. After patients without malignancies as determined in preoperative specimens were excluded, the concordance rate was 90.7% (127/140). We found a better accuracy for the diagnosis of SQC than for that of ADC (89.3% vs. 80.4%, p = 0.012), which was also in agreement with previous studies [13,20]. On the other hand, the sensitivity for ADC was slightly better than that for SQC (75.7% vs. 65.6%, p = 0.138), indicating that ADC seems to have little oversight by preoperative specimens.

IHC studies are usually required if obvious morphological features of squamous or glandular differentiation are not identified. Similar to the findings of this study, prior studies found that ~75% of bronchial NSCLC samples may be subtyped using morphology alone, and 93% of all NSCLC cases may be subtyped when combined immunostaining [21]. Other studies also demonstrated that only a low population (6%) of cases remained without a probable subtype after morphologic examination and IHC [22]. The low not-typed rate in these studies suggests that we should perform IHC as a routine utilization for subtyping of difficult cases.

Several factors affect the diagnostic accuracy. The frequency of diagnostic errors induced by some of these factors, including the biopsy size and degree of differentiation has been described [20]. In our study, the tumor size was correlated with the sensitivity and concordance rate: the larger the diameter, the greater the sensitivity and cell type agreement.

Until date, therapeutic decisions are heavily dependent on the histological subtype of lung cancer (SQC vs. non-SQC) and its molecular characteristics (e.g., *EGFR* mutation and ALK rearrangement states). Furthermore, many previous studies showed the expression and functions of proteins and genes in lung cancer, and it may be significantly related to tumor progression and metastasis [23-26].

Both *EGFR* mutation and *ALK* rearrangements are almost exclusively seen in ADC. According to multiple large phase III crinical trials [11,27-29], the new classification recommends that all patients with advanced lung ADC be tested for *EGFR* mutation, as *EGFR* tyrosine kinase inhibitors can be used as first-line chemotherapy for patients with *EGFR* mutations. Crizotinib has been approved by the US Food and Drug Administration (FDA) for advanced ADC with *ALK* rearrangements [30]. Therefore, testing for *EGFR* mutations and *ALK* rearrangements in patients with advanced ADC is no longer only a research tool, and should be performed in clinical practice. On the other hand, several molecular targets such as *FGFR1* amplification and *DDR2* mutation have been discovered in SQC [31,32]. Target therapies that might be beneficial in this selected subpopulation have never been reported. These results indicate that misdiagnosis of ADC as SQC in preoperative specimens should be avoided so as to prevent the loss of opportunity to select beneficial molecular-targeted therapy for these patients in clinical practice. Although such a result was not seen, there were three (1.3%) misclassifications between ADC and SQC in the preoperative specimens, and in all, SQC was diagnosed as ADC. Fortunately, there was only a lone patient who might have lost the opportunity to receive EGFR-TKI therapy by missed diagnosis (0.44%, shown as case report).

Our retrospective study had some limitations. First, only half of the patients who underwent surgery received preoperative diagnosis by TBB or CTNB. Second, there may be variability between intra- and inter- pathologist in lung cancer diagnosis. Because a smaller amount of tissue is obtained, the diagnostic assessment might be more difficult and more inconsistencie in the diagnosis of pathologists might occur. In such cases, multidisciplinary approach and expert consultation are recommended [33].

## Conclusions

In conclusion, our study suggested that the concordance rate for the diagnosis of NSCLC subtypes, especially "squamous" or "non-squamous" histology, between preoperative and surgical specimens was satisfactory as compared with other reports. Pretreatment diagnosis of lung cancer using small samples is reasonable for selection of the optimal treatment. However, our case report presented above serves to emphasize the need to be aware that diagnosis based on preoperative small samples might be different from that made from surgical specimens, so as to not lose the opportunity for effective treatment.

## Consent

Written informed consent was obtained from the patient for the publication of this report and any accompanying images.

## Competing interests

The authors declare that they have no competing interests.

## Authors’ contributions

TY and KS designed and wrote the paper. NO, HY, NT and MO participated in the design of the study and reviewed the literature. KS and MN performed surgery. All authors read and approved the final manuscript.

## References

[B1] Jemal A Bray F Center MM Ferlay J Ward E Forman D Global cancer statistics CA Cancer J Clin 2011 61 69 90 21296855 10.3322/caac.20107

[B2] Crino L Weder W Van Meerbeeck J Felip E Early stage and locally advanced (non-metastatic) non-small-cell lung cancer. ESMO clinical practice guidelines for diagnosis, treatment and follow-up Ann Oncol 2010 21 103 1 10.1093/annonc/mdq207 20555058

[B3] Itaya T Yamaoto N Ando M Ebisawa M Nakamura Y Murakami H Asai G Endo M Takahashi T Influence of histological type, smoking history and chemotherapy on survival after first-line therapy in patients with advanced non-small cell lung cancer Cancer Sci 2007 98 226 230 17233840 10.1111/j.1349-7006.2006.00379.x PMC11158360

[B4] Travis WD Brambilla E Noguchi M Nicholson AG Geisinger KR Yatabe Y Beer DG Powell CA Riely GJ Van Schil PE Garg K Austin JH Asamura H Rusch VW Hirsch FR Scagliotti G Mitsudomi T Huber RM Ishikawa Y Jett J Sanchez-Cespedes M Sculier JP Takahashi T Tsuboi M Vansteenkiste J Wistuba I Yang PC Aberle D Brambilla C Flieder D International association for the study of lung Cancer/American thoracic Society/European respiratory society international multidisciplinary classification of lung adenocarcinoma J Thorac Oncol 2011 6 244 285 21252716 10.1097/JTO.0b013e318206a221 PMC4513953

[B5] Shah PL Singh S Bower M Livni N Padley S Nicholson AG The role of transbronchial fine needle aspiration in an integrated care pathway for the assessment of patients with suspected lung cancer J Thorac Oncol 2006 1 324 327 17409878

[B6] Einhorn LH First-line chemotherapy for non-small-cell lung cancer: is there a superior regimen based on histology? J Clin Oncol 2008 20 3485 3486 18506022 10.1200/JCO.2008.17.2056

[B7] Stinchcombe TE Grilley-Olson JE Socinski MA If histology matters J Clin Oncol 2010 28 1810 1812 20212244 10.1200/JCO.2009.27.1247

[B8] Scagliotti GV Parikh P Von Pawel J Biesma B Vansteenkiste J Manegold C Serwatowski P Gatzemeier U Digumarti R Zukin M Lee JS Mellemgaard A Park K Patil S Rolski J Goksel T De Marinis F Simms L Sugarman KP Gandara D Phase III study comparing cisplatin plus gemcitabine with cisplatin plus pemetrexed in chemotherapy-naïve patients with advanced-stage non–small-cell lung cancer J Clin Oncol 2008 26 3543 3551 18506025 10.1200/JCO.2007.15.0375

[B9] Lynch TJ Bell DW Sordella R Gurubhagavatula S Okimoto RA Brannigan BW Harris PL Haserlat SM Supko JG Haluska FG Louis DN Christiani DC Settleman J Haber DA Activating mutations in the epidermal growth factor receptor underlying responsiveness of non–small-cell lung cancer to gefitinib N Engl J Med 2004 350 2129 2139 15118073 10.1056/NEJMoa040938

[B10] Paez JG Jänne PA Lee JC Tracy S Greulich H Gabriel S Herman P Kaye FJ Lindeman N Boggon TJ Naoki K Sasaki H Fujii Y Eck MJ Sellers WR Johnson BE Meyerson M EGFR mutations in lung cancer: correlation with clinical response to gefitinib therapy Science 2004 304 1497 1500 15118125 10.1126/science.1099314

[B11] Mok TS Wu YL Thongprasert S Yang CH Chu DT Saijo N Sunpaweravong P Han B Margono B Ichinose Y Nishiwaki Y Ohe Y Yang JJ Chewaskulyong B Jiang H Duffield EL Watkins CL Armour AA Fukuoka M Gefitinib or carboplatin-paclitaxel in pulmonary adenocarcinoma N Engl J Med 2009 361 947 957 19692680 10.1056/NEJMoa0810699

[B12] Johnson DH Fehrenbacher L Novotny WF Herbst RS Nemunaitis JJ Jablons DM Langer CJ DeVore RF 3rd Gaudreault J Damico LA Holmgren E Kabbinavar F Randomized phase II trial comparing bevacizumab plus carboplatin and paclitaxel with carboplatin and paclitaxel alone in previously untreated locally advanced or metastatic non-small-cell lung cancer J Clin Oncol 2004 22 2184 2191 15169807 10.1200/JCO.2004.11.022

[B13] Edwards SL Roberts C McKean ME Cockburn JS Jeffrey RR Kerr KM Preoperative histological classification of primary lung cancer: accuracy of diagnosis and use of the non-small cell category J Clin Pathol 2000 53 537 540 10961178 10.1136/jcp.53.7.537 PMC1731233

[B14] Lindeman NI Cagle PT Beasley MB Chitale DA Dacic S Giaccone G Jenkins RB Kwiatkowski DJ Saldivar JS Squire J Thunnissen E Ladanyi M Molecular testing guideline for selection of lung cancer patients for EGFR and ALK tyrosine kinase inhibitors: guideline from the College of American Pathologists, international association for the study of lung cancer, and association for molecular pathology J Thorac Oncol 2013 8 823 859 23552377 10.1097/JTO.0b013e318290868f PMC4159960

[B15] Cataluna JJ Perpina M Greses JV Calvo V Padilla JD París F Cell type accuracy of bronchial biopsy specimens in primary lung cancer Chest 1996 109 1199 1203 8625667 10.1378/chest.109.5.1199

[B16] Matsuda M Horai T Nakamura S Nishio H Sakuma T Ikegami H Tateishi R Bronchial brushing and bronchial biopsy: comparison of diagnostic accuracy and cell typing reliability in lung cancer Thorax 1986 41 475 478 3024348 10.1136/thx.41.6.475 PMC460368

[B17] Rudd RM Gellert AR Boldy DA Studdy PR Pearson MC Geddes DM Bronchoscopic and percutaneous aspiration biopsy in the diagnosis of bronchial carcinoma cell type Thorax 1982 37 462 465 6291189 10.1136/thx.37.6.462 PMC459342

[B18] Clee MD Duguid HL Sinclair DJ Accuracy of morphological diagnosis of lung cancer in a department of respiratory medicine J Clin Pathol 1982 35 414 419 7076869 10.1136/jcp.35.4.414 PMC497672

[B19] Payne CR Hadfield JW Stovin PG Barker V Heard BE Stark JE Diagnostic accuracy of cytology and biopsy in primary bronchial carcinoma J Clin Pathol 1981 34 773 778 6267108 10.1136/jcp.34.7.773 PMC493813

[B20] Arinç S Saltürk C Ertuğrul M Sulu E Tuncer L Nergis S Selvi U Cell type agreement between bronchoscopic biopsy and thoracotomy specimens in primary lung cancer Tuberk Toraks 2007 55 378 382 18224506

[B21] Loo PS Thomas SC Nicolson MC Fyfe MN Kerr KM Subtyping of undifferentiated non-small cell carcinomas in bronchial biopsy specimens J Thorac Oncol 2010 5 442 447 20195168 10.1097/JTO.0b013e3181d40fac

[B22] Sigel CS Moreira AL Travis WD Zakowski MF Thornton RH Riely GJ Rekhtman N Subtyping of non-small cell lung carcinoma: a comparison of small biopsy and cytology specimens J Thorac Oncol 2011 6 1849 1856 21841504 10.1097/JTO.0b013e318227142d

[B23] Shi Y Wu H Zhang M Ding L Meng F Fan X Expression of the epithelial-mesenchymal transition-related proteins and their clinical significance in lung adenocarcinoma Diagn Pathol 2013 8 89 doi:10.1186/1746-1596-8-89 23706092 10.1186/1746-1596-8-89 PMC3671218

[B24] Zheng S Du Y Chu H Chen X Li P Wang Y Ma Y Wang H Zang W Zhang G Zhao G Analysis of MAT3 gene expression in NSCLC Diagn Pathol 2013 9 8 166 doi:10 24107548 10.1186/1746-1596-8-166 PMC3853379

[B25] Xiong Y Bai Y Leong N Laughlin TS Rothberg PG Xu H Nong L Zhao J Dong Y Li T Immunohistochemical detection of mutations in the epidermal growth factor receptor gene in lung adenocarcinomas using mutation-specific antibodies Diagn Pathol 2013 8 1 27 Epub ahead of print 23419122 10.1186/1746-1596-8-27 PMC3635899

[B26] Shilo K Wu X Sharma S Welliver M Duan W Villalona-Calero M Fukuoka J Sif S Baiocchi R Hitchcock CL Zhao W Otterson GA Cellular localization of protein arginine methyltransferase-5 correlates with grade of lung tumors Diagn Pathol 2013 8 201 doi:10.1186/1746-1596-8-201 24326178 10.1186/1746-1596-8-201 PMC3933389

[B27] Maemondo M Inoue A Kobayashi K Sugawara S Oizumi S Isobe H Gemma A Harada M Yoshizawa H Kinoshita I Fujita Y Okinaga S Hirano H Yoshimori K Harada T Ogura T Ando M Miyazawa H Tanaka T Saijo Y Hagiwara K Morita S Nukiwa T North-East Japan Study Group Gefitinib or chemotherapy for non-small-cell lung cancer with mutated EGFR N Engl J Med 2010 362 2380 2388 20573926 10.1056/NEJMoa0909530

[B28] Mitsudomi T Morita S Yatabe Y Negoro S Okamoto I Tsurutani J Seto T Satouchi M Tada H Hirashima T Asami K Katakami N Takada M Yoshioka H Shibata K Kudoh S Shimizu E Saito H Toyooka S Nakagawa K Fukuoka M West Japan Oncology Group Gefitinib versus cisplatin plus docetaxel in patients with non-smallcell lung cancer harbouring mutations of the epidermal growth factor receptor (WJTOG3405): An open label, randomised phase 3 trial Lancet Oncol 2010 11 121 128 20022809 10.1016/S1470-2045(09)70364-X

[B29] Rosell R Carcereny E Gervais R Vergnenegre A Massuti B Felip E Palmero R Garcia-Gomez R Pallares C Sanchez JM Porta R Cobo M Garrido P Longo F Moran T Insa A De Marinis F Corre R Bover I Illiano A Dansin E De Castro J Milella M Reguart N Altavilla G Jimenez U Provencio M Moreno MA Terrasa J Muñoz-Langa J Erlotinib versus standard chemotherapy as first-line treatment for European patients with advanced EGFR mutation-positive non-small-cell lung cancer(EURTAC): A multicentre, open-label, randomized phase 3 trial Lancet Oncol 2012 13 239 246 22285168 10.1016/S1470-2045(11)70393-X

[B30] Kwak EL Bang YJ Camidge DR Shaw AT Solomon B Maki RG Ou SH Dezube BJ Jänne PA Costa DB Varella-Garcia M Kim WH Lynch TJ Fidias P Stubbs H Engelman JA Sequist LV Tan W Gandhi L Mino-Kenudson M Wei GC Shreeve SM Ratain MJ Settleman J Christensen JG Haber DA Wilner K Salgia R Shapiro GI Clark JW Anaplastic lymphoma kinase inhibition in non-small-cell lung cancer N Engl J Med 2010 363 1693 1703 20979469 10.1056/NEJMoa1006448 PMC3014291

[B31] Dutt A Ramos AH Hammerman PS Mermel C Cho J Sharifnia T Chande A Tanaka KE Stransky N Greulich H Gray NS Meyerson M Inhibitor-sensitive FGFR1 amplification in human non-small cell lung cancer PLoS One 2011 6 e20351 21666749 10.1371/journal.pone.0020351 PMC3110189

[B32] Hammerman PS Sos ML Ramos AH Xu C Dutt A Zhou W Brace LE Woods BA Lin W Zhang J Deng X Lim SM Heynck S Peifer M Simard JR Lawrence MS Onofrio RC Salvesen HB Seidel D Zander T Heuckmann JM Soltermann A Moch H Koker M Leenders F Gabler F Querings S Ansén S Brambilla E Brambilla C Mutations in the DDR2 kinase gene identify a novel therapeutic target in squamous cell lung cancer Cancer Discov 2011 1 78 89 22328973 10.1158/2159-8274.CD-11-0005 PMC3274752

[B33] Kayser K Fritz P Drlicek M Rahn W Expert consultation by use of telepathology–the Heidelberg experiences Anal Cell Pathol 1995 9 53 60 7577755

